# Medical and Philosophical Intelligence

**Published:** 1812-05

**Authors:** 


### MEDICAL AND PHILOSOPHICAL INTELLIGENCE.

A PAPER by Mr. Brodie, entitled "*Further experiments and observations on the action of certain poisons on the animal system,*" was read at the Royal Society, on Thursday, February 27th.

In this paper, Mr. Brodie first detailed some experiments made with the view of recovering animals laboring under the effects of the worrara poison, by means of artificial respiration. In one of them, them, when this process had been continued for an hour and a half, the animal began to give signs of life, and ultimately recovered.

The rest of the paper was occupied by an account of the effects produced by different mineral poisons. Those of arsenic, muriate of barytes, and emetic tartars, were found to be the same, whether the poisons were applied to wounded surfaces, or given internally. From some circumstances, the author is led to conclude that none of these poisons produce their effects, even on the stomach, till they are absorbed into the circulation.

The action of these poisons was found to be less simple than that of vegetable poisons. The author considers the inflammation of the stomach produced by arsenic to be rarely the cause of death, which he attributes, in the majority of instances, to the action of the poison on the heart and brain, which are affected nearly in an equal degree.

The effects of the emetic tartar and muriate of barytes were found to be very similar to those of arsenic, except that the heart was affected in a less degree. The circulation might be kept up after death had apparently taken place by inflating the lungs, but only for a limited period of time, and in no instance was the animal restored to life.

The paper concludes with some observations on the effects of corrosive sublimate, which, when taken in large doses, is said by its chemical action to destroy the texture of the internal coat of the stomach, before it enters the circulation, in this respect differing from the mineral poisons which were employed in the other experiments.

---

We have been favored, by Dr. George Pearson, with the following statement, and a specimen of the plant mentioned in it. It was communicated to him by Mr. Derbishire, of Maddox-street, who had it from a gentleman of Gray's-inn, (Mr. Chipman,) who himself had received it direct from North America.

"I accidentally have heard of a plant that grows wild in this country in great abundance, called the *Bone-set*, which I almost suspect to be the basis of this medicine (L'Eau Medicinale). Its qualities are, that it is intensely *bitter;* upon being taken as an herb tea, it uniformly excites *nausea*, and, according to the state of the stomach and intestines, operates either as a powerful *emetic* or *cathartic*. It is considered by the people as an universal restorative, and is taken in almost all complaints, and considered as a sovereign remedy for them all, and particularly good in rheumatic complaints. This plant has, I find, been long known in the United States, and perhaps under some other name in England."—4th *March*, 1811.

"I have some thoughts of sending a parcel of the *Bone-set*, ripened in my own garden, after being transplanted from its native bed of *upland-swamp* in Gage-Town, of course requiring to be kept well watered, which I took care should be done. It has seeded, and possibly may be propagated in England from those seeds, if they should be sufficiently ripened. I shall send the plant with the seeds seeds on it as gathered from the garden. I have no idea *now* that it is the basis of the Eau Med. (Dr. Moore having settled that point to my full satisfaction); but it may prove an useful addition to the London Pharmacopœia, if not already known there. It is said certainly to have the qualities I formerly mentioned, and is universally used by the people in this country as a cure for all *chronic* complaints. If it is really good for any thing, its virtues will soon be discovered in the medical laboratory of that seat of literature and arts for which it is destined. It is esteemed here a very great stomachic, and very useful in rheumatic and bilious complaints, and in these cases has proved at least a very innocent medicine."—12th *December*, 1811.

On comparing the specimen sent to us with another in the herbarium of Aylmer Bourke Lambert, esq. we are enabled to say positively that it is the *Eupatorium perfoliatum*. It is not new, however, in this country; it is to be found growing, we believe, in many gardens in the neighbourhood of the metropolis, and was cultivated in the Chelsea garden as early as 1699. (*Hort. Kew. from Morrison.*) As it has always been considered to be a medicinal plant by the aborigines as well as the Anglo-Americans, we perceive the reason for its early introduction. The plants of any country that first attract attention are those which are believed to cure diseases; the high character of the *E. perfoliatum* in its native country, would recommend it to the first botanists who visited North America.

We understand Mr. Derbishire is endeavoring to ascertain its medicinal powers; and we recommend him to inquire for it in the gardens, where probably he will procure a supply that may enable him to extend his investigation. While, however, he is looking to a foreigner for an addition to our materia medica, we hope he will not overlook a native of this family, the *Eupatorium cannabinum*, a plant possessing very active qualities; and which it may not be unreasonable to suppose may be the basis of the *Eau Medicinale.* The whole of the *E. cannabinum* is intensely bitter; and it vomits and purges briskly. It is stated to be a rough medicine, and to be only used with caution. It is reported to cure dropsy, jaundice, and scurvy. By this last is probably meant, in the incorrect and popular sense, some herpetic disease of the skin. The active properties of the *E. cannabinum* demand attention. Medical botany should look to the whole of this numerous genus. The Bone-set of America, and the Hemp Agrimony of Europe, are known to be active substances; but what are the other forty-eight or fifty species of this family?

---

The Jacksonian prize of the Royal College of Surgeons, in London, for the year 1811, has been adjudged to Mr. Joseph Hodgson, of Bucklersbury, London, for his Dissertation on the Diseases of the Arteries and Veins, comprising the Pathology and Treatment of Aneurism and wounded Arteries.

---

*Dr. Stokes* is employed on a Botanical Materia Medica, to make four octavo volumes.

The

The experiments of Professor Leslie to produce *ice* by evaporation in the air-pump, have been varied and extended in France, by Messrs. Clement and Desormes. They have proposed to apply the evaporation in vacuo, on a large scale, to the drying of gunpowder which, being done without fire, will not be attended with danger. The French chemists are endeavoring to apply the evaporation in vacuo to the drying and preserving fruit and vegetables.

In consequence of these processes carrying on in France, Professor Leslie has addressed a letter to the Caledonian Mercury, in proof of his discovery, the theory and practice of which he demonstrated in London last year, when he freely explained the various important uses to which the principle might be applied, *particularly to the introduction of the apparatus into hospitals in sultry climates.* Since the Professor's return to Edinburgh, we are informed he has improved his coolers, so as to produce *ice* in large quantities, by much inferior powers to those he first used.

---

Mr. Brown, surgeon of his Majesty's ship Fylla, has invented an instrument for *compressing the Subclavian Artery.* This instrument manifests great ingenuity in its construction, and we have the authority of the following certificates to shew that the employment of it fully answers the intended object.

"Gentlemen, *Royal Hospital, Haslar, Dec.* 7, 1811.

"We duly received your letter of the 22d ult. enclosing one from Mr. Brown, surgeon of the Fylla; and, having examined and tried the screw instrument therein described, are of opinion that it effects the purpose for which it is intended, namely, that of compressing the subclavian artery, as it passes over the first rib, and completely stopping the pulsation at the wrist.

"We think, therefore, that it may be a very useful instrument in amputations at the articulation of the humerus, in cases where no steady medical assistant can be procured; and where, from the motion of the ship, or any other cause, an assistant cannot steady himself sufficiently to depend upon the compression he could otherwise accomplish with his thumb. We are, &c.

"CHA. DODS.
"J. STEPHENSON.
"DN. M'ARTHUR."

"Approved. CHARLES CRAVEN, Governor.

"*To the Commissioners for Transports, &c.*"

"*Royal York Hospital, Chelsea, Jan.* 21, 1812.

"We have tried, at this hospital, the instrument for compressing the subclavian artery, invented by surgeon Brown, of his Majesty's ship Fylla, and are of opinion that it will completely answer the purpose for which it is intended, and that it may be advantageously employed both in the naval and military service.

"J. PRICE, M.D. Physician to the Forces.
"CHA. GRIFFITH, Surgeon to the Forces.
"GEO. ALBERT, Surgeon to the Forces."

*Theatre*

*Theatre of Anatomy.*—Lectures on Anatomy, Physiology, Pathology, and Surgery, by Mr. John Taunton, F.A.S. Member of the Royal College of Surgeons of London, Surgeon to the City and Finsbury Dispensaries, City of London Truss Society, &c.—In this course of Lectures it is proposed to take a comprehensive view of the structure and economy of the living body, and to consider the causes, symptoms, nature, and treatment, of surgical diseases, with the mode of performing the different surgical operations; forming a complete course of anatomical and physiological instruction, for the medical or surgical student, the artist, and the professional or private gentleman. An ample field for professional edification will be afforded, by the opportunity which pupils may have of attending the clinical and other practice of both the City and Finsbury Dispensaries. The summer course will commence on Saturday, May the 23d, 1812, at eight o'clock in the evening precisely, and be continued every Tuesday, Thursday, and Saturday, at the same hour.

---

Dr. Ramsbotham will commence a summer course of Lectures on the Science and Practice of Midwifery, on Monday, May the 11th, at his house, No. 9, Old Jewry.

---

Dr. Clutterbuck will begin his summer course of Lectures on the Theory and Practice of Physic, Materia Medica, and Chemistry, on Monday, June the 8th, at ten o'clock in the morning, at his house, No. 1, in the Crescent, New Bridge-street. The different lectures are given on the alternate days through the week: viz. Theory and Practice of Physic, on Mondays, Wednesdays, and Fridays; Materia Medica and Chemistry, on Tuesdays, Thursdays, and Saturdays, at the same hour.

---

Dr. Adams will deliver his summer course of Lectures at his house in Hatton-garden. The particulars will be given in our next Number.

---

*Theatre of Anatomy, Blenheim-street, Great Marlborough-street.*—The summer course of Lectures on Anatomy, Physiology, and Surgery, will be commenced on Saturday, the 6th of June, at seven o'clock in the morning, by Mr. Brookes.—Anatomical converzationes will be held weekly, when the different subjects treated of will be discussed familiarly, and the students' views forwarded: to these none but pupils can be admitted. Spacious apartments, thoroughly ventilated, and replete with every convenience, are open at five o'clock in the morning, for the purposes of dissecting and injecting, where Mr. Brookes attends to direct the students, and demonstrate the various parts as they appear on dissection. The inconveniences usually attending anatomical investigations, are counteracted by an antiseptic process. Pupils may be accommodated in the house. Gentlemen established in practice, desirous of renewing their anatomical knowledge, may be accommodated with an apartment to dissect in privately.

Cure

*Cure from the Bite of the Snake, Coluber Naga, Fort St. George, Madras.*—I am induced to give the following history of the situation of a Sepoy bit by a Coluber Naga, as I think it may be useful in shewing how much is within the power of early assistance to preserve life in all cases where the bite of that dangerous snake may be on the extremities, and when on any other part of the body, that the farther progress of danger will be arrested, if the part bit is cut out while there is a chance of recovery.

On the 30th ultimo, in the afternoon, Syed Mahomet, a Sepoy, about 38 years of age, one of the guard at Mr. Cassamajor's house, was brought to me with two ligatures drawn tight round his arm, one near the shoulder, the other a little above the elbow; he told me he had been bit by a Cobra Capello in the outer part of his left hand between the little finger and the wrist, where a small wound appeared, from which a little blood oozed; that he was asleep at the time, but was instantly roused by an acute pain, darting up his arm; the Sepoys on guard immediately applied the two ligatures I have mentioned, and being shewn the broken part of the wall near to which their companion was bit, they searched and found a Cobra Capello, which they killed and brought to me; it was a half-grown Coluber Naga, between three and four feet long.

More than half an hour from the accident had elapsed before I saw him; he then laboured under extreme pain in the whole hand and forearm to a little above the elbow, where the first ligature was applied. From so much time having elapsed, which would have proved fatal had not the ligatures been applied, I was doubtful of success, from the poison having extended its influence so far; but as there was no other affection of the body, except what was raised by the acute pain from the fingers to below the second ligature, I was induced to cut out the piece bit at even this late period, and immediately immersed the whole left arm in warm water; the wound bled freely, and as he felt relief by compressing the arms with both hands, and compressing it always from the elbow to the wrist, this was continued in warm water for above half an hour, when I had the gratification to find that the acute pain had descended to near the wound; I then ventured to remove the two ligatures on the arm, and continued the immersion in warm water for above half an hour longer, without any tendency to ascension of pain; I therefore gave him two glasses of brandy in water, and 60 drops of laudanum, to relieve him from a sense of bulk and weight in his hand, more distressing than acute pain, and causing a tendency to syncope; ordered a large poultice to the hand, and I desired him to inform me if any acute pain should again dart up from the wound, or further than the elbow. There was then no tension or swelling except a little on the back of the hand; but the uneasy sensation I have mentioned continued in the evening, and which I did not expect would subside for some time.

31st.—Slept none all night from a throbbing lancinating pain all over his hand and forearm up to where the lowest ligature was applied, plied, the whole swelled and tense, with a considerable increase of heat—ordered the poultices to the wound to be frequently repeated; he slept a little in the day-time, and ate victuals. Afternoon—As the swelling, tension, and heat, of the forearm and hand had considerably increased, with the lancinating throbbing pain up to the elbow, accompanied with quick full pulse, severe head-ache, and other febrile symptoms, which seemed to threaten a serious termination if a check was not put to the progress of rapid inflammatory action—with this view ten leeches were applied where there was greatest pain, and the bleeding from their bites encouraged by fomentation; considerable relief was thus obtained, and a complete intermission from fever took place in the evening—gave him some brandy and water, as he complained of weakness.

1st April.—No tension now on the forearm, swelling diminished, but there is still a lancinating throbbing pain to a little above the elbow where the first ligature was applied; he had a recurrence of fever about midnight, a very short cold stage, a hot fit for about four hours, and profuse sweating for about an hour longer; a complete intermission then took place, and the fever in a similar form again recurred to-day at noon. For the relief of pain five more leeches were applied, and with success; finding the fever of an intermittent nature, though of an universal form, I ordered bark.

2d.—Fever recurred last night, as before, about midnight, but without any sensible cold stage, and ceased with sweating about five in the morning—skin this morning cool, pulse 84, and rather weak—he vomited his bark last night—is hungry, and desirous of food—throbbing lancinating pain gone, only a little soreness on the back of the hand and fingers, which are swelled, and the swelling has a firm feel—ordered the bark to be given in brandy and water. Evening, took five drams of bark, which did not disagree with his stomach—fever however returned about midnight, but of a much shorter duration, and followed by a more profuse perspiration—no sleep from pain near the elbow, where the second ligature had been applied, and throbbing lancinating pain on the back of the hand and fingers, and particularly the joints, where the firm swelling continues. Some relief obtained by fomentation—took his bark as before—no fever to-day at noon;—as so much benefit had been obtained by leeches, five more were applied, two to the elbow and three to the back of the hand—ordered the bark to be taken till midnight.

4th.—No fever, but he was prevented from sleeping by a throbbing pain in his fingers, stretching up to the back of his hand; his fingers, and particularly their joints, still swelled and hard—ordered more leeches, as nothing seems more effectual in removing the suffusion produced by the action of the poison and the throbbing pain connected with it. The patient at his own request had ten leeches applied to his fingers and knuckles, by which he says the pain in his hand, the only remaining pain, is entirely removed. As he complains of weakness, ordered him some brandy and water.

5th.—No fever or pain; great weakness in the muscles of the hand or forearm, and the wound does not yet put on a healthy suppurating surface. The leeches applied to the forearm have all been in a line from the wound to the elbow, showing the pain there to have been greatest, from the patient having pointed out the places. Sent home as out of danger.

This case I consider interesting, as it shows the action of the poison of the Coluber Naga, where vital parts are not primarily affected; and, as such an opportunity of knowing it but seldom occurs, I have thought proper to give it in detail.

The observations to be drawn I think are, that this man's life was saved by the early application of a ligature, and to the arm where there being only one bone, a sufficient compression to prevent the further influence of the poison could be made.

2d.—That the excision of the part bit, by a removal of the poison instilled into the wound, prevented any farther action; for at no time was there any distress beyond the ligature above the elbow.

3d.—From the time that had elapsed before excision, and the full influence of the poison as far as the ligature above the elbow admitted, that it is probable the poison of the Coluber Naga is not absorbed into the system, but acts on the nerves; for, if absorption had taken place, its further progress, on the removal of the ligatures, could not have been prevented, and would have proved fatal; and which probably shows the advantage of cutting out the bite at all times.

4th.—From there being no tension or swelling excepting on the back of the hand, and none followed by the application of the two ligatures, the intermediate part of the arm between them, the distress that occurred from swelling, tension, heat, throbbing, and lancinating pain, must all be considered characteristic of the action of the poison, and probably also the form of the fever; the utility of the exhibition of the bark may thus be doubted, the fever ceasing with the cessation of the cause.

5th.—That the inflammatory action induced seems analogous to what occurs in membranous inflammation, and proceeded with that activity as at one time to threaten sphacelus, had not the free application of leeches and abstraction of blood by subsequent fomentations checked the increased excitement: showing strongly the activity and power of even the influence of the poison that had been communicated from the wound, for the poison itself I consider to have been removed by the excision.

(Signed) A. BERRY.

---

*Cure of Ascites, in which the Operation of Tapping was performed through the Vagina.*—Mrs. Warren, of Brierley Hill, near Dudley, a middle-aged woman, underwent the operation of tapping in the month of July, 1810. The peritoneum was punctured through the linea semilunaris, but, the disease still continuing, the operation was repeated in the following October, and again in March last. The abdomen became distended a fourth time, and a tumor appeared in the vagina from the pressure of the fluid upon the inflected peritoneum neum between the vagina and rectum. This protrusion gradually increased, and caused the peritoneum to have a depressed appearance. On the 28th of July, 1811, the patient being placed upon the edge of a bed with her knees elevated, I attempted to introduce into the cavity of the peritoneum through the protrusion a large trocar and canula; but in this I at first failed, for when the least pressure was applied, the tumor receded. Being determined, however, to do the operation on this depending part, I introduced the two fore-fingers of my left hand into the vagina beyond the tumor, and by pressing downwards I kept it stationary, and thus succeeded in passing the trocar in a direction upwards and forwards. On withdrawing it the water followed with uncommon velocity, and before the canula was withdrawn the abdomen appeared completely emptied. The wound made by the instrument was healed in three days. The patient bore it better and felt less inconvenience after this operation than either of the preceding. She is now in as good health as usual, but is of a a habit which precludes the expectation of her complete recovery.

---

*Case of extensive Fracture of the Skull, with Wound of the Meningeal Artery.*—Thomas Whyatt, of Walton, near Clent, 15 years of age, on the 5th of August, 1811, received a kick from a horse upon his right temple. About four hours after the accident, when he was first seen, he was senseless, his pupils were dilated and his pulse feeble. The integuments on the side of the head which had received the injury were so much swoln that it was impossible to ascertain whether the skull was fractured or not. The symptoms, however, of pressure upon the brain were so evident, that it was judged necessary to ascertain whether this was produced by a depressed portion of bone in the situation of the external injury. An incision was accordingly made a little above the orbit, and continued across the temporal muscle towards the occiput about six inches. This incision was intersected by a second in the course of the fibres of the temporal muscle continued to the zygoma, which had been broken by the accident. The muscle was then detached and turned back with the scalp, when a great extent of bone was found to be broken and depressed, consisting of a portion of the frontal bone, the anterior and inferior part of the parietal bone, part of the squamous portion of the temporal bone, and the upper part of the greater ala of the sphenoid bone. The trephine was applied upon the parietal bone near the fractured edge, and, after elevating the depressed portion and removing the broken fragments, there was a profuse hæmorrhage from the meningeal artery which had been lacerated by the accident. The bleeding, however, soon ceased, by sponging the part with cold water, and did not recur. The dura mater was not injured, but it did not regain its level when the depressed bone was removed. The flaps and temporal muscle were carefully applied to the dura mater where the bone had been removed, and were retained by a ligature and adhesive straps. The functions of the brain were speedily restored; the antiphlogistic plan was rigidly pursued, and the boy recovered without a bad symptom, notwith-

 standing standing the removal of a very extensive portion of the skull. The ligature was removed on the 5th day, when the wound had nearly united. In less than a month he returned to his labour, and felt no inconvenience from the accident. He is now capable of moving his lower jaw with the utmost facility, although the temporal muscle is attached merely to the dura mater.

*Case of the good Effects of Stramonium in Asthma.*—Mr. J. C., a medical gentleman, 42 years of age, of middle stature and full habit, had been afflicted with a cough and difficulty of breathing for several years. About four years ago he was attacked with distinct paroxysms of asthma, which came on in the usual manner and progressively increased, so much so that he could not lie down without the greatest dread of suffocation. This fit continued three or four weeks and then left him. In a few months it again attacked him, and for three years afterwards he had regular paroxysms, with great difficulty of respiration in the intervals. During this time he took various medicines suggested both by himself and numerous physicians of eminence; and, in short, during three years, made an adequate trial of every article in the Materia Medica recommended for this complaint, but without deriving the least benefit. About 12 months ago, during a violent paroxysm, he commenced smoking the stramonium, and after using one pipe-full found the symptoms wonderfully relieved, and by repeating it once or twice a day the paroxysm entirely subsided. He is now occasionally attacked in the night, but by rising and smoking one pipe of the stramonium the difficulty of breathing generally ceases immediately, and when this is not the case he is so much relieved that he can lie down and sleep with comparative comfort. Since he first had recourse to this remedy he has had no regular fit of asthma; and his breathing gets so much better, that he is of opinion that by perseverance in the remedy he shall entirely recover. It does not affect his stomach, nor his head, but seems entirely to act on the organs of respiration.

*Case of Femoral Hernia, in which the Intestine sphacelated.*—On the 22d of February, 1811, I was requested to visit Mrs. Stokes, of Ratcliff, near Nottingham, an aged woman, who had for some years been afflicted with a femoral hernia, which had now been strangulated six or eight days. During this time she had no evacuation from her bowels, and was now in the most excruciating pain, and suffering all the symptoms of strangulated hernia. I made a gentle attempt to reduce the protruded bowel, but, this proving ineffectual, and the strangulation having existed so long, I judged it prudent not to allow any further delay, and immediately proposed the operation as an only resource, having indeed but little hopes of its success from the violence of the symptoms and the advanced period of strangulation. My patient, however, readily submitted, and I commenced the operation in the usual manner. Upon opening the sac the intestine came into view; it was greatly discolored, but did not appear at the time in a state of sphacelation. It was firmly connected nected to the sac by adhesions which I separated, and having dilated the stricture I returned the bowel into the cavity of the abdomen. She was much relieved by the operation, and in a few hours had a regular evacuation, which was frequently repeated during the three following days. On the fourth day I dressed the wound. The bowel had then protruded afresh, and it was in a mortified condition. In one place it was ruptured, and afforded a free discharge of fæces. I immediately removed about three inches of the sphacelated bowel, and left the remainder out of the abdomen. The wound was regularly dressed, but the whole surface of it sloughed, and the fæces were incessantly discharged from it for the space of five and thirty days. At the end of this period the parts put on a healthy appearance, and the granulations began to diminish the opening into the intestine. At length it was completely closed, and the fæces was discharged in the natural manner. About the beginning of May she enjoyed perfect health. The wound had completely healed, and she suffered no symptoms of her former complaint.

---

*Case of Hydrophobia.*—A child, aged four years, was bitten by a dog on the 10th of March, 1811. The wound, which was situated at the inner angle of the eye-lid, was very slight, and soon healed, without affording any subsequent inconvenience. On the 29th of the same month she complained of tooth-ache, to which she had been liable; she passed a restless night with frequent sighings, and on the following morning was attacked with a convulsive action of the throat, and great agitation of her whole frame: the symptoms increased, she became feverish, and the sight of fluids produced the greatest disgust. These symptoms continued during the second and third days, without much increase: the skin was hot and dry, the pulse very frequent, and no evacuation had taken place from the bowels for a week. The difficulty of respiration, with great anxiety and sighings, were excited by the presence of liquids, or a stream of air. In the evening of the third day, *two* scruples of calomel and three grains of opium, suspended in a little mucilage, were injected into the stomach through an elastic gum tube; an aloetic clyster was also administered; this was attended with a temporary aggravation of the spasms, which afterwards subsided to their former state. During the evening she repeatedly asked for liquids, which, when offered, excited the greatest horror; the eyes became dull and heavy, with an appearance of inflammation, the paroxysms became more frequent and violent, the extremities more cold, and she was insensible: the approach of a candle excited convulsions, she spit out a quantity of mucus, and expired early on the morning of the fourth day. Upon dissection no morbid appearances were discovered, except an inflammation of the trachea and bronchia, which contained a quantity of puriform fluid.—*Lond. Med. Rev.*

---

Lately died, at Dunmow, in Essex, in his 92d year, Dr. Robert Courthorpe Sims. He took the degree of doctor of physic, at the university of Edinburgh, in 1744, and wrote on the occasion an inaugural dissertation, *De Vomica Pulmonis:* but, being more desirous of being really useful than of acquiring riches or fame, he was content to practise as a surgeon and apothecary in the small country town where he died. His hours of relaxation were chiefly spent in his garden, in the cultivation of which he took the greatest delight, particularly in varying the arrangement of the walks, the grass, and the plantations, so as to change the general form of the whole, with the view of producing picturesque effect. In these alterations he was generally allowed to shew much taste; and Dr. Sims's garden, though limited in extent to about an acre, was admired beyond any other in the neighbourhood, and not unfrequently excited the curiosity of strangers: to himself it afforded a perpetual source of innocent amusement for upwards of sixty years. In 1807 he published a small tract, entitled, "An Essay on the Constitution of Man, natural, moral, and religious." The design of this work was especially to attempt to impede the torrent of that irreligious philosophy which has been spread over Europe. The improvement and republication of this essay, was his chief employment till his death.

A few weeks since, died at the house of his father-in-law, Lawrence Peel, esq. of Ardwick, Mr. Benjamin Gibson, one of the surgeons of the Manchester Infirmary. Though cut off at an early period of life, yet he had long enjoyed such a share of the public confidence as is seldom bestowed, except on greater length of days and more matured experience. By nature he was liberally endowed with gifts, that peculiarly fitted him for a profession in which excellence can only result from the union of great delicacy of the bodily senses with strength and sagacity of intellect. These endowments he had improved, by an ardent and persevering study of the branches of science more immediately connected with his vocation; and he united to them a gentleness and urbanity of manner, arising from the most humane feelings, which, while they led to all due sympathy with his suffering patients, never interfered with the steady execution of his professional resolves. But it was not only by the successful practice of what he had learned from others, that Mr. Gibson was distinguished. Influenced by an honorable ambition, he aimed at the higher object of extending the usefulness of his profession, and of rendering it, in some diseases, a more perfect instrument of relief. The discoveries which his talents enabled him to make, he freely communicated; for no man ever more cordially despised empirical conduct, whether manifested by the ostentatious display of knowledge, or by its illiberal concealment. Consistently with this part of his character, he employed himself, at a period of his lingering illness when the vigor of his mind still survived the wreck of his bodily strength, in bequeathing to the world his improvements in the treatment of some of the diseases of the eye, in the cure of which he had been singularly fortunate. The work, united to his general eminence as a surgeon, has procured him much more than a local reputation; and will deliver his name to posterity as the improver of an art, which, while living, he adorned by his high attainments; by enlarged views of its objects and dignity; and by the benevolent extension of its relief, with an equal hand, to the poor and to the affluent.

